# Rapid and Ultrasensitive Colorimetric Biosensors for Onsite Detection of *Escherichia coli* O157:H7 in Fluids

**DOI:** 10.1021/acssensors.3c02339

**Published:** 2024-02-06

**Authors:** Bofeng Pan, Ahmed Y. El-Moghazy, Makela Norwood, Nitin Nitin, Gang Sun

**Affiliations:** † Biological and Agricultural Engineering, University of California, Davis, California 95616, United States; ‡ Department of Food Science and Technology, University of California, Davis, California 95616, United States

**Keywords:** foam-based ELISA, *Escherichia coli* O157:H7, on-site detection, foodborne pathogens, colorimetric biosensor

## Abstract

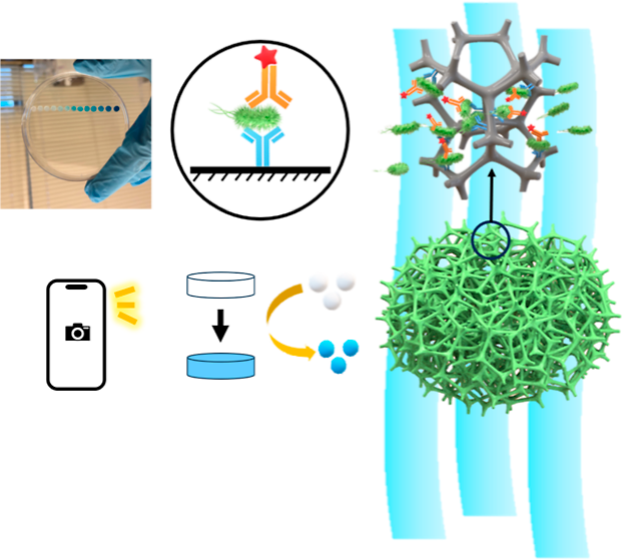

This study presents a breakthrough in the field of onsite bacterial detection, offering an innovative, rapid, and ultrasensitive colorimetric biosensor for the detection of *Escherichia coli* (*E. coli*) O157:H7, using chemically modified melamine foam (MF). Different from conventional platforms, such as 96-well plates and fiber-based membranes, the modified MF features a macroporous reticulated three-dimensional (3D) framework structure, allowing fast and free movement of large biomolecules and bacteria cells through the MF structure in every direction and ensuring good accessibility of entire active binding sites of the framework structure with the target bacteria, which significantly increased sensitive and volume-responsive detection of whole-cell bacteria. The biosensing platform requires less than 1.5 h to complete the quantitative detection with a sensitivity of 10 cfu/mL, discernible by the naked eye, and an enhanced sensitivity of 5 cfu/mL with the help of a smartphone. Following a short enrichment period of 1 h, the sensitivity was further amplified to 2 cfu/mL. The biosensor material is volume responsive, making the biosensing platform sensitivity increase as the volume of the sample increases, and is highly suitable for testing large-volume fluid samples. This novel material paves the way for the development of volume-flexible biosensing platforms for the record-fast, onsite, selective, and ultrasensitive detection of various pathogenic bacteria in real-world applications.

Foodborne illnesses represent significant public health challenges worldwide.^1^ Among these, *Escherichia coli* O157:H7 is a particularly concerning pathogen because of its low infectious dose and severe health consequences.^2,3^ This specific serotype of *E. coli* can cause diseases varying from diarrheal illness to hemorrhagic colitis and hemolytic uremic syndrome, which can lead to kidney failure or death in extreme cases. *E. coli* O157:H7 was reported of causing an estimated 63,000 hemorrhagic colitis illnesses annually in the United States.^4,5^ Its low infectious dose and high pathogenicity and a potential risk of its contamination in water and food sources make it a significant threat to food safety and public health.^6,7^ Currently, the detection of the existence of *E. coli* O157:H7 in food and water samples has relied heavily on conventional methods including culture-based assays, polymerase chain reaction (PCR), and isothermal amplification.^8−11^ While these methods have proven to be effective over time, they possess several limitations. The culture-based assay, with its high reliability and sensitivity, is considered the gold standard in the field of bacterial detection.^12^ However, it is time-intensive (2–3 days) and requires highly trained personnel, making it unsuitable for rapid onsite detection.^13^ PCR’s exceptional sensitivity is counterbalanced by its need for expensive equipment and complex preparation procedures.^14,15^ Isothermal amplification methods amplify DNA at a consistent temperature, contrasting the temperature cycling of PCR. While adept at detecting pathogens in trace amounts, this method can be hindered by complex primer design and contamination risks, potentially leading to a high false-positive rate.^16^ Other methods, including flow cytometry, gas chromatography, Fourier transform infrared spectroscopy (FTIR), Raman spectroscopy, etc., also require professional tools, being both costly and time-consuming.^17,18^ The existing diagnostic methods prove challenging to implement in low-income countries, where high mortality rates prevail due to a lack of adequate diagnostic tools.^19,20^ Therefore, there has been a pressing need for a more efficient, affordable, and rapid detection method to combat this public health threat.

Currently, paper-based colorimetric biosensors have been reported as being capable of detecting pathogenic bacteria in food and water with naked eyes.^21,22^ The straightforward design and operation of the paper-based ELISA (p-ELISA) colorimetric sensing systems make them an appealing choice for on-site detection systems, which are disposable and potentially operatable by untrained personnel.^23−27^ However, a variety of technical challenges restrict their use in assessing food safety, in terms of microbial contamination. Detection of a small number of pathogenic bacteria within a large volume of a food or water sample has proven difficult using these conventional systems because of their relatively low detection sensitivity. Additionally, the complexity of food matrices—including the existence of fats, proteins, saccharides, fibers, and various salts—can significantly affect the separation of target bacteria from the other contents in food samples and the subsequent color development reaction. A key factor contributing to the limitations of p-ELISA is the layered fiber mat structures of the papers and fibrous membranes formed during the manufacturing processes, making the sensor media heterogeneous in their vertical direction from other directions. The inconsistency in the direction significantly impedes the penetration of large biomolecules through the p-ELISA media, leading to less incorporation of biomolecules on fiber surfaces inside the media in comparison to the outside surfaces.^28^ Even though the media are often described as having a three-dimensional (3D) structure, the inner part of the materials is seldom fully utilized. Particularly when whole cells of microorganisms are used as antigens, their micrometer sizes restrict them from diffusing and penetrating into the media and even if they manage to traverse the layered narrow porous structure, they often become trapped and are difficult to wash off through the system.^29^ This structural characteristic may cause inhomogeneous colorimetric signals, strong sample matrix effects, and high false-positive rates of the sensors, additionally reducing the sensitivity of p-ELISA sensors fabricated using filter papers and nitrocellulose or nanofibrous membranes (NFs).^30^ Hence, an ideal medium for ELISA bacteria biosensors is conceptualized as a homogeneous, 3D, and macroporous structure, enabling unrestricted bacterial cell migration in every direction.

In our previous studies, we demonstrated that foam-based ELISA (f-ELISA) using melamine foam (MF) as a medium offers unique advantages.^31^ It was proven to be rapid, sensitive, additive, and volume-responsive across different types of approaches, including direct, competitive, and sandwich ELISA by detecting the SARS-CoV-2 spike protein and chloramphenicol (CAP). In this context, we believe that f-ELISA is even better suited for *E. coli* O157:H7 detection, given the larger size of bacteria compared to chemical compounds and proteins. The application of f-ELISA in the detection of bacterial cells could fully demonstrate the advantages of the macroporous features offered by the chemically modified MF. In contrast to conventional ELISA (c-ELISA), which is restricted by the limited surface area of a 96-well plate and other p-ELISA methods, bacteria as antigens can move freely in every direction within this macroporous 3D matrix. This enhanced freedom of movement facilitates an amplified interaction between the immobilized antibodies and antigens, leading to substantial enrichment and heightened sensitivity in colorimetric detection. The testing process needs less than 1.5 h to complete both preparation and detection, and the results indicated that the sensors made of the modified MF materials can detect *E. coli* O157:H7 at a level of 10 cfu/mL by the naked eye with a limit of detection (LOD) at 5 cfu/mL when supplemented by a smartphone. Following a brief enrichment period of 1 h, the sensitivity was further amplified to 2 cfu/mL. Interestingly, the sensitivity increases as the volume of the sample increases, making this sensing material highly suitable for testing large-volume fluid samples, such as milk, drinking fluids, agricultural water, etc. In essence, this study paves the way for a rapid, sensitive, and volume-flexible biosensing platform, using *E. coli* O157:H7 as a proof of concept, which holds promise for the rapid and ultrasensitive detection of various pathogenic bacteria in real-world applications.

## Experimental Section

### Materials

*N*, *N*′-disuccinimidyl carbonate (DSC), triethylamine (TEA), 1,4-dioxane, acetone, phosphate-buffered saline (PBS), 3,3′,5,5′-tetramethylbenzidine (TMB), and 96-well plates were purchased from Thermo Fisher Scientific. *E. coli* O157 mouse anti-*E. coli* monoclonal antibody (Ab-*E. coli*) and *E. coli* rabbit anti-*E. coli* polyclonal (HRP) antibody (Ab-*E. coli*-HRP) were purchased from Lifespan Biosciences (Shirley, MA, USA). MFs were purchased from Swisstek (Brewster, NY, USA). Maximum recovery diluent (MRD) was purchased from Sigma-Aldrich (St. Louis, MO, USA). PBS, tryptic soy broth (TSB), and tryptic soy agar (TSA) were purchased from Fisher Scientific (Fair Lawn, NJ, USA). All other chemicals were of analytical grade and were supplied by Merck (Darmstadt, Germany). Rifampin-resistant *E. coli* O157:H7 (ATCC700728), *E. coli* BL21 (ATCC BAA-1025), and *Listeria innocua* (ATCC 33,090) were obtained from ATCC (Manassas, VA, USA). MacConkey agar was supplied by Difco (Sparks, MD, USA). SYBR Green I nucleic acid stain (10 × % concentrate) was purchased from Invitrogen (Carlsbad, CA, USA). Programmable syringe pump was purchased from NewEra Instruments (Suffolk County, NY, USA).

Morphologies of all MF-based samples were analyzed using a scanning electron microscope (Quattro ESEM, Thermo Scientific). Electronic micrometer thickness gauge (Neoteck) was used to measure the thickness of the MF membranes. An ultraviolet–visible spectrophotometer (UV–vis, Evolution 600, Thermo Fisher) was used to measure the absorbance of the bacteria solution. Laser scanning confocal microscopy (Olympus FV1000) was used to obtain fluorescence microscopy images. A light panel (5″ × 4″, LP-100N) was employed to provide consistent lighting for capturing images of the sample results.

### Bacterial Culture and Sample Preparation

An overnight *E. coli* O157:H7 culture was developed and transferred by using a loop from stored bacteria TSA plates, which were prepared following a reported method,^32^ into 10 mL of sterile TSB, followed by incubation at 37 °C with constant shaking at 200 rpm. After incubation for 16 h, the *E. coli* O157:H7 culture reached a concentration of 10^9^ cfu/mL. This overnight culture was centrifuged at 13,000 rpm for 1 min to harvest the bacterial cells, which were washed twice and resuspended in sterile PBS. The *E. coli* O157:H7 suspension (10^9^ cfu/mL) was then diluted in PBS to achieve varying bacterial concentrations.

### Measurement of Diffusion of Bacteria

The diffusions of *E. coli* O157:H7 through the MFs in three different thicknesses, nitrocellulose paper (NP), and NF were measured using a side-by-side diffusion chamber (PermeGear Co.).^29^ MF membranes at different thicknesses (1–3 mm), as well as NP and NF, were separately placed between the two chambers, which were tightly sealed in a water bath with a temperature of 25 °C. To prewet the membranes, each chamber was filled with 3 mL of a PBS solution for 15 min. Following this, 3 mL of *E. coli* O157:H7 suspension (10^7^ cfu/mL) was injected into the donor chamber. Stirring bars were set in both chambers, operating at a speed of 750 rpm throughout the tests. At regular time intervals, 1 mL of the sample solution was extracted from each chamber and added back to the chambers after the measurement via UV–vis at the wavelength of 600 nm.^33^ The concentration of *E. coli* O157:H7 was determined by UV–vis spectroscopy, based on calibration curves provided in the Supporting Information (Figure S1). The subsequent analysis of bacterial concentration in the receptor chamber over increasing time intervals allowed for an assessment of the diffusion properties of the bacteria through the MF membranes.

### Vertical Flow Test through Materials

The vertical flow test was carried out by separately placing MF membranes at different thicknesses (1–3 mm), NP and NF in the bottom of a 20 mL syringe, creating a filtration column (Figure S2). A 1 mL *E. coli* O157:H7 suspension at a concentration of 10^4^ cfu/mL was passed through the columns containing the different testing matrices. The collected filtrates were serially diluted and plated for bacterial counting using TSA containing 0.05 g/L rifampicin, which allowed the growth of the inoculated rifampicin-resistant *E. coli* O157:H7 with prevention contamination from the tested matrices. After passing 10 mL sterile PBS to replace any remaining bacterial solutions, the different membranes were transferred into a sterile centrifugal tube (15 mL size) containing 1 mL of a releasing buffer (MRD with 0.01% lecithin), which were incubated for 2 min and then vortexed vigorously for 1 min to recover any captured bacterial cells.^32^ The quantification of the recovered bacterial cells was performed by the same serial dilution and plate counting method using TSA (containing 0.05 g/L rifampicin).

### Foam-based ELISA Platform Preparation

A typical chemical structure of MFs is shown in Figure S3. The chemical modification processes of MF were the same as those reported in a previous publication (Figure S4a).^31^ The MF samples were all in circular form of 1.0 mm thickness and 5.0 mm diameter. The structures of MF and DSC-modified MF membrane (NHS@MF) samples were characterized by FTIR spectroscopy with the same spectra as those reported in the literature (Figure S4b).^31^ Then, 100 μL of Ab-*E. coli* solution (10 mg/L) was added to the NHS@MF membranes and incubated for 30 min at room temperature. After antibody immobilization, the remaining active sites were blocked using 200 μL of 3% skim milk (SKM), and we defined the material as Ab@NHS@MF.

### Foam-based ELISA in *E. coli* O157:H7 Detection

The analytical performance of the biosensing platform-based MF (f-ELISA) was evaluated by adding 200 μL of *E. coli* O157:H7 at varied concentrations (ranging between 0 and 10^7^ cfu/mL) to the f-ELISA membranes. The incubation process continued for 30 min with mild shaking. After incubation, any unbound bacteria were removed by washing with PBS buffer. Subsequently, 100 μL of Ab-*E. coli*-HRP (1 mg/L) was applied to each membrane. After incubation for 20 min, the membranes were washed with a PBST solution (PBS plus 0.05% v/v tween-20), followed by a rinse with PBS buffer and subsequent air drying. Next, 35 μL of TMB substrate was added onto the membranes, and color images of the membranes were obtained following the method in the literature.^31^

In order to further investigate the impact of the sample volume on *E. coli* O157:H7 detection, varied volumes of the bacterial samples (from 100 μL to 10 mL) were employed in experiments following the above protocol.

To ensure the long-term efficacy and repeatability of the f-ELISA system, we examined the stability and activity of stored antibodies on the modified MF. The Ab@NHS@MF membranes were treated using 10% sucrose as a stabilizer followed by freeze-drying.^34,35^ They were stored at a consistent temperature of 4 °C and assessed over a duration of 90 days. At predetermined time intervals, aliquots were retrieved and utilized in the f-ELISA assay to detect *E. coli* O157:H7 following the same protocols.

### Fluorescence Images of *E. coli* O157:H7 Cells Captured by Ab@NHS@MF

*E. coli* O157:H7 cells harvested from centrifuging 1 mL of its overnight culture at 13,000 rpm for 1 min were washed twice and resuspended with sterile PBS. A 100 μL portion of 10x SYBR green I was added and incubated in the dark for 5 min. Afterward, the labeled bacterial cells were recovered, washed with sterile PBS three times to remove the excess SYBR green I dye, and resuspended with sterile PBS. The labeled *E. coli* O157:H7 suspension was diluted in PBS to obtain a cell concentration of 10^5^ cfu/mL. Then, 100 μL of *E. coli* O157:H7 (10^5^ cfu/mL) was added to the Ab@NHS@MF membranes and allowed for incubation for 30 min under mild shaking. Afterward, the membranes were washed with PBST and then with PBS buffer, followed by air-drying. A laser scanning confocal microscope (Olympus FV1000) with a tetramethylrhodamine-isothiocyanate (TRITC ex 541 nm/em 572 nm) filter was employed to acquire fluorescence images of the SYBR green I labeled *E. coli* O157:H7, with the assistance of image processing software ImageJ to convert the acquired fluorescence image files from TIFF into JPG.^36^

### Detection of *E. coli* O157:H7 in Real Samples

An irrigation water sample was collected from the Campbell Tract at the University of California, Davis, the Solano County District agricultural irrigation water system (Agwater). The Agwater sample was autoclaved to remove any background noise created by the Agwater. Then, the autoclaved Agwater sample was spiked with *E. coli* O157:H7 in a concentrations range of 10–10^4^ cfu/mL. A nonspiked autoclaved Agwater sample was employed as a control. For analysis of the samples using f-ELISA, the Ab@NHS@MF membrane was mounted into a syringe needle pocket, and 5 mL of the prepared sample solution was loaded into a 20 mL syringe and flowed through the filtering media in the needle at a controlled flow rate of 10 mL/h using a syringe pump (NewEra Instruments, USA). Moreover, the nonautoclaved Agwater sample was tested without spiking using the f-ELISA, and the achieved results were compared with the plate counting assay approach using a selective medium (MacConkey agar plates). The presence of red colonies on the MacConkey agar plates indicates the presence of *E. coli* O157:H7 in the Agwater sample.

### Colorimetric Data Processing

Upon the addition of the TMB substrate to the Ab@NHS@MF membranes, they were positioned inside an LED light box and on a light panel (5 in. × 4 in., LP-100N) with an illuminance of 12,000 lx. Images were taken using a smartphone camera (iPhone 14pro max) fixed at 50 cm above the membranes. The intensity of the color was represented by the red channel (*R*) from the RGB values obtained from Adobe Photoshop (2022) following eq 1.^37^
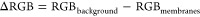
1where RGB_background_ represents the *R* value of the white background (without HRP), and RGB_membranes_ is the *R* value of the Ab@NHS@MF membranes.

### Statistical Analysis

All assays for the study were performed in triplicate. The data are presented as mean ± standard deviations (SD). Intergroup comparisons were analyzed using Student’s *t*-test (two-tailed). The levels of significance were defined as *P < 0.05, **P < 0.01, and ***P < 0.001.

The linear relationship between the observed and predicted values was assessed using the correlation coefficient (*R*). A *P* value of less than 0.05 was considered statistically significant. All statistical analyses were conducted using GraphPad Prism version 8.0.2.

## Results and Discussion

### Filtering and Diffusion Test of Bacteria in MF Membranes

**Figure 1:**
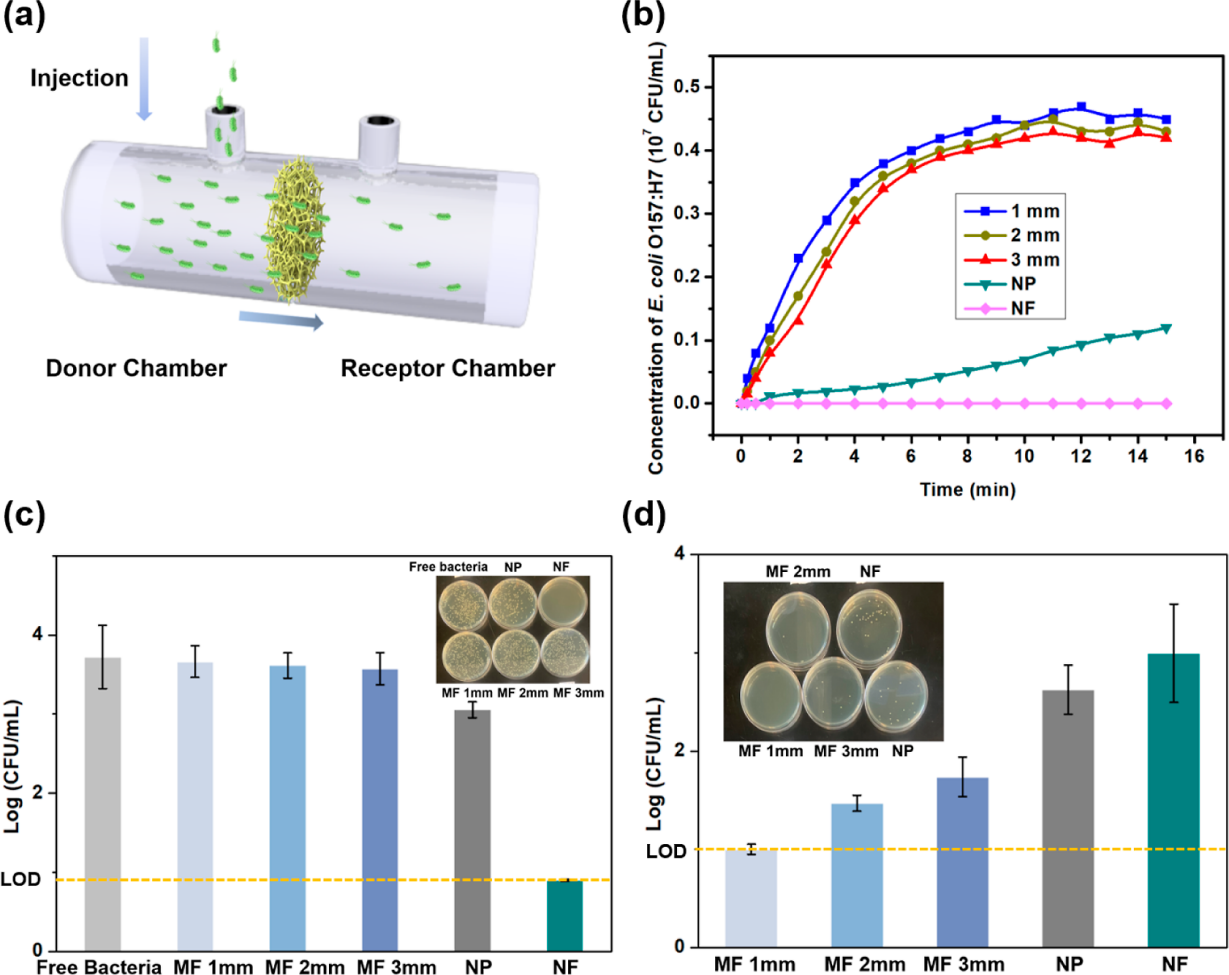
(a) Scheme of the used side-by-side chamber. (b) Correlation between diffusion time and concentration of *E. coli* O157:H7 diffused through NF, NP, and MF in different thicknesses. (c) Vertical flow test of *E. coli* O157:H7 solution (at 10^4^ cfu/mL concentration) through various materials: NF, NP, and MF with different thicknesses, with each material positioned at the base of a syringe. (d) Unspecific adsorption of *E. coli* O157:H7 onto the tested materials after buffer wash (NF thickness = 0.21 mm; NP thickness = 0.18 mm). Data are shown as mean ± SD, based on *n* = 3 independent experiments. *LOD = limit of detection.

Diffusion of large molecules and particles in chartaceous materials is heterogeneous and notably slow in vertical directions of the materials because of the layered fiber mat structure and substantially decreased effective pore sizes.^24^ However, the homogeneous 3D macroporous framework structure of the MF materials in all directions enables the penetration of large biomolecules or particles moving around with minimal resistance. Based on our previous studies,^31^ we found that compared to the diffusion performances of the biomolecules of various sizes (40–150 kDa) in NFs and NPs, which need several hours to achieve a steady state of diffusion, the thicknesses of the MF materials and sizes of protein molecules did not exhibit a noticeable difference, which could be considered negligible if the duration of interaction between MF and the substrates extended beyond 10 min. However, the diffusion properties of bacteria in the macroporous MF materials could be different, as bacterial cells are in significantly larger dimensions than proteins and other biomolecules—with lengths ranging from 1 to 10 μm and widths between 0.2 and 1 μm.^38^ Thus, as depicted in Figure 1a, a side-by-side diffusion chamber was utilized to investigate the diffusion behaviors of *E. coli* O157:H7, aligning with our focus on this bacterial strain in subsequent experiments using the innovative f-ELISA system. The concentration changes of *E. coli* O157:H7 in the receiver chamber represent the bacterial cells that have diffused through the MF materials with thicknesses of 1.0, 2.0, and 3.0 mm, or through NF and NP, respectively, for a duration of 18 min, and the results are plotted in Figure 1b. In both NFs [poly(vinyl alcohol-*co*-ethylene), PVA-*co*-PE] and NPs, the diffusion of *E. coli* O157:H7 required several hours to attain a steady state. Conversely, in all MF membranes, regardless of their thickness, steady-state diffusion was achieved in less than 11 min. The variation in concentrations observed in the MF membranes with different thicknesses could be attributed to the fact that the increased thickness in the MF corresponds to a greater material volume. This increased volume can potentially retain more of the bacterial solution, resulting in a slightly reduced concentration in the receptor chamber upon reaching a steady state. The diffusion test simulates the process of the MF membranes encountering bacterial solution samples under a specific stirring rate. As evidenced by the results, the highly porous and homogeneous framework structure and macropore size of the MF materials allow for easy penetration of whole bacterial cells through the media with minimum resistance. This facilitates the thorough exposure of the MF’s 3D framework to pathogens in liquid form, substantially augmenting the likelihood of interactions between the immobilized antibody and *E. coli* O157:H7, increasing the sensitivity in pathogen detection of the f-ELISA media. A liquid filtering test was also conducted to evaluate the potential application of the MF media in additive filtering sensing devices (Figure S2). As shown in Figure 1c, a minimal amount of *E. coli* O157:H7 was trapped when the solution flowed through a 1 mm thick MF membrane, and only slight increases in trapped bacteria cells were observed for the 2 and 3 mm thick MF membranes. Both NF and NP could trap or block more bacterial cells with significantly reduced concentrations of the bacteria shown in the filtered solutions. The nonspecific adsorption results of each medium after buffer wash were consistent with the vertical flow test (Figure 1d). Besides, the nonspecific binding of *E. coli* O157:H7 on the MF membranes of varying thicknesses (1–3 mm) was investigated, and the results revealed that while the 1 mm thick MF membrane showed no measurable nonspecific bacterial binding, the 2 and 3 mm membranes, due to their increased volumes, retained relatively more bacteria solutions, as evidenced in Figure 1d, which highlights the use of 1 mm MF in the sensors. In contrast, NP and NF samples exhibited a significant retention of the bacteria. This retention could elevate the background in biosensors using these two materials as detection platforms, potentially increasing the false-positive rate and reducing the sensitivity of the assay. Therefore, the 1 mm thick MF was selected for subsequent tests to optimize the accuracy and sensitivity of the f-ELISA for *E. coli* O157:H7 detection.

### Capture of *E. coli* O157:H7 Based on Ab@NHS@MF

**Figure 2:**
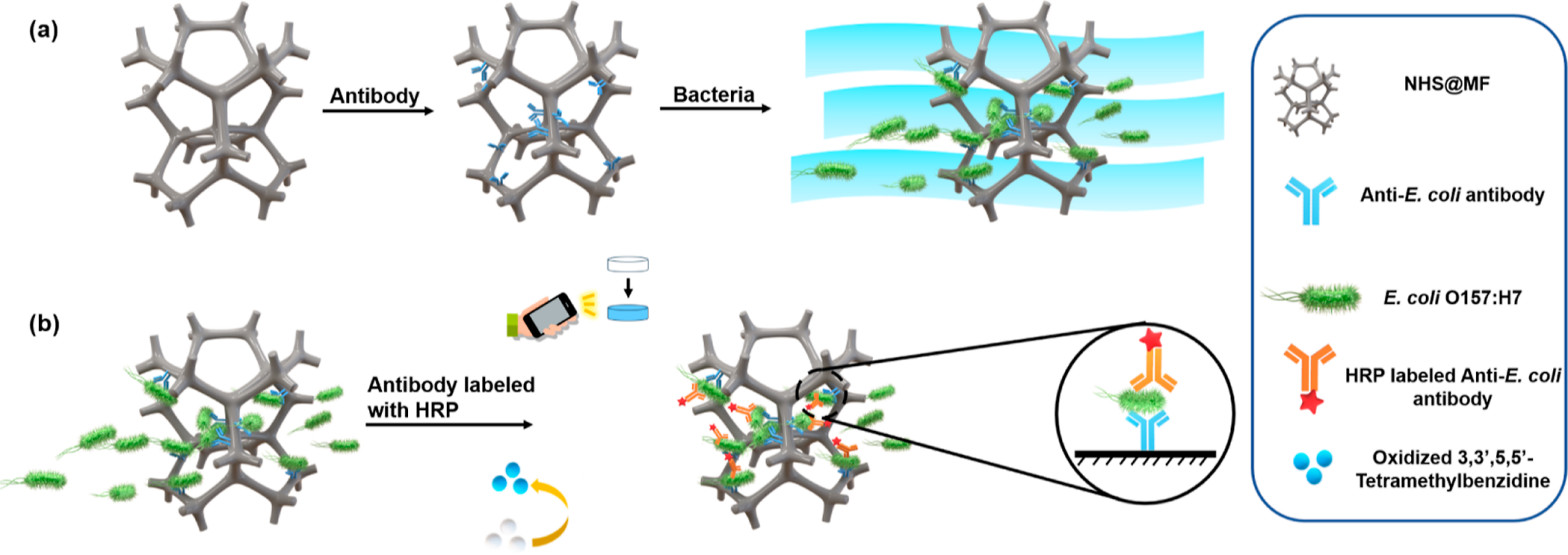
Schematic illustration of the preparation and use of foam-based sandwich ELISA (f-ELISA). (a) Immobilization of antibodies and capture of bacteria. (b) Addition of HRP-labeled secondary antibody and enzymatic substrate TMB to generate color signals and obtaining of images using a smartphone.

**Figure 3:**
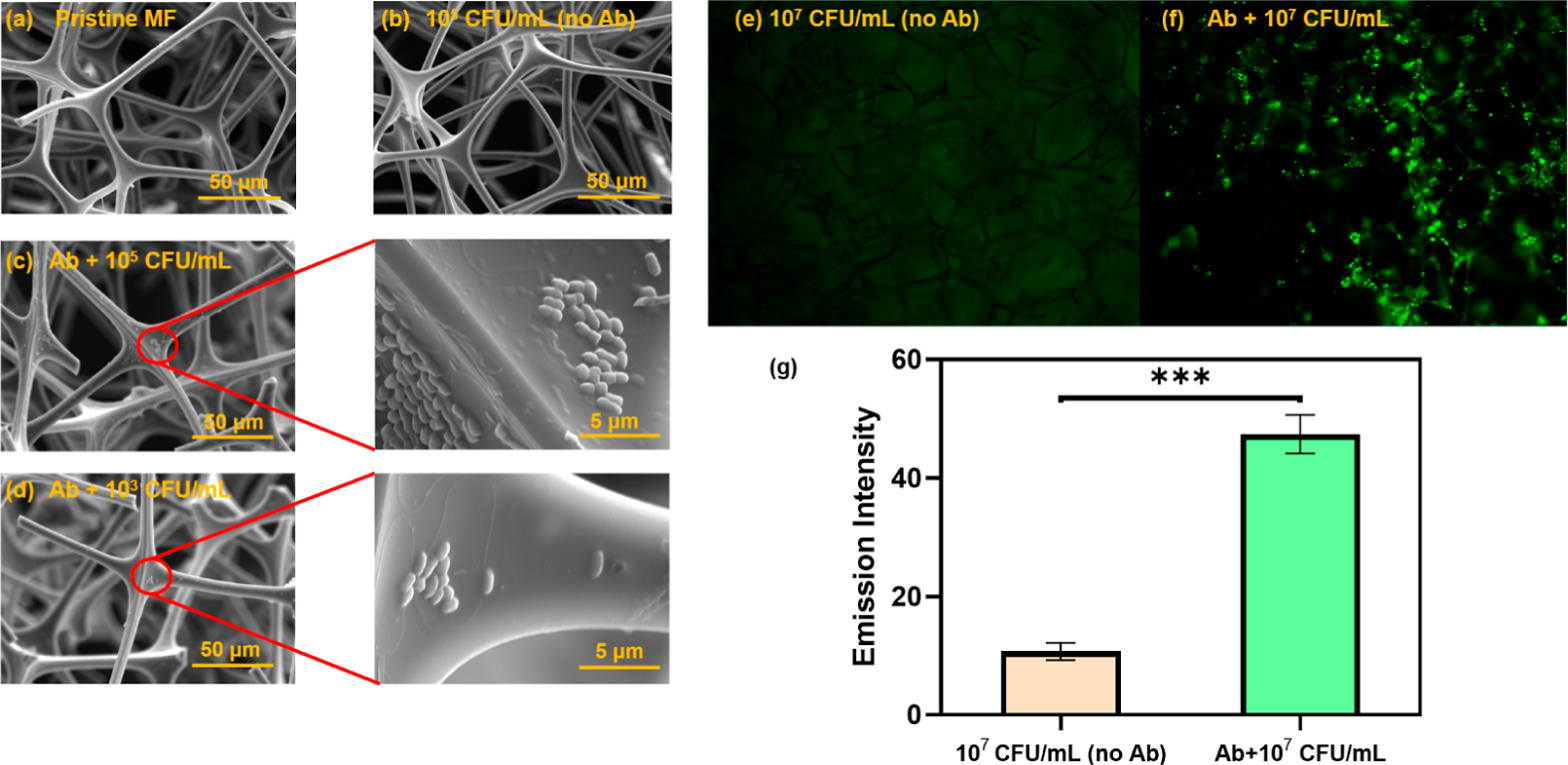
SEM images of (a) pristine MF, (b) NHS@MF after incubation with *E. coli* O157:H7 solution (10^5^ cfu/mL), (c) Ab@NHS@MF incubated with *E. coli* O157:H7 solution (10^5^ cfu/mL), and (d) Ab@NHS@MF after incubation with *E. coli* O157:H7 solution (10^3^ cfu/mL). Fluorescence microscopic images of (e) NHS@MF and (f) Ab@NHS@MF, after incubation with *E. coli* O157:H7 solution at a concentration of 10^7^ cfu/mL. Data are shown as mean ± SD, based on *n* = 3 independent experiments. ***P < 0.001 (two-tailed Student’s *t*-test).

As our previous results indicated, the covalent immobilization of proteins onto the MF was achieved by chemically modifying the secondary amino groups of the melamine polymer following the reported method (Figure S3).^31^ The chemical modification and protein immobilization reactions on the MF are depicted in Figure S4a. The successful incorporations of the reactive *N*-hydroxysuccinimide (NHS) groups to the MF and formation of NHS@MF were confirmed with the band of 1730 cm^–1^ in the FTIR spectra (Figure S4b).^39^ The antibody loading capacity on the NHS@MF exceeded the capacities on both NHS@NF and NP, when compared by mass, resulting in considerably high number of interactive sites for target bacterial cells than those on the materials used in p-ELISA sensors.^31^ After the immobilization of Ab- *E. coli*, Ab@NHS@MF should be able to capture the target bacteria specifically from the liquid samples as illustrated in Figure 2a. From SEM characterization results, it is evident that the morphology of the MF framework structures remains unchanged after chemical modification, protein immobilization, and bacteria capture (Figure 3a–c). As demonstrated in Figure 3b, in the absence of immobilized antibodies on the material, no unspecific binding was observed. This suggests a low background in subsequent f-ELISA tests, corroborating the results from the diffusion and vertical flow tests mentioned earlier. Furthermore, as shown in Figure 3c,d, it is very clear that when the Ab@NHS@MF membranes were used in the capture of *E. coli* O157:H7 in varied concentrations (10^3^ and 10^5^ cfu/mL), the amounts of the bacteria used and captured on the MF media were related. From the results of the fluorescence microscopy, after being modified with Ab- *E. coli*, blocked with SKM, and incubated with *E. coli* O157:H7 solution at a concentration of 10^7^ cfu/mL, the difference in signal intensities of NHS@MF and Ab@NHS@MF is statistically significant, and the green dots, representing the bacterial cells, homogeneously distribute through the framework of the entire membrane (Figure 3e–g). Given these attributes, f-ELISA could be a highly promising platform for detecting whole-cell antigens.

### Analytical Performance of f-ELISA

The f-ELISA sensing system, rooted in its novel approach for detecting whole-cell antigens, showed great potential in the initial experiments. In the following section, we detail the performance metrics of this innovative system, focusing on its specificity and sensitivity. The general f-ELISA procedure of detecting *E. coli* O157:H7 is shown in Figure 2. In the presence of the Ab- *E. coli* immobilized on NHS@MF, *E. coli* O157:H7 is recognized and then captured by the MF-based sensor media. Then, the addition of Ab-*E. coli*-HRP to the system and its reaction with TMB lead to the generation of colorimetric signals. By analyzing the colorimetric signals of the f-ELISA obtained from the pictures taken by a smartphone (Figure 2b), the detection of bacteria can be achieved on-site.

First, the optimization of experimental conditions, including the concentrations of antibody and HRP and the reaction time of HRP with TMB substrate, was conducted, with the results shown in Figure S5. The optimal concentrations of Ab-*E. coli* and Ab-*E. coli*-HRP were identified as 5 and 2 mg/L, respectively, and a reaction time of 6 min was selected for the interaction between TMB substrate and HRP. To attest the specificity of the assay, we carried out an array of control experiments, including the tests without Ab-*E. coli*-HRP, Ab-*E. coli*, SKM, or target, respectively. The data from these controls, shown in Figure S6, further confirmed the robustness of the f-ELISA sensor.

Besides the negative control experiments that did not utilize HRP, there was either no color development or there was a minimal color response in scenarios where Ab-*E. coli* was absent, as illustrated in Figure S6, aligning perfectly with the results of SEM and fluorescence microscopy (Figure 3).

**Figure 4:**
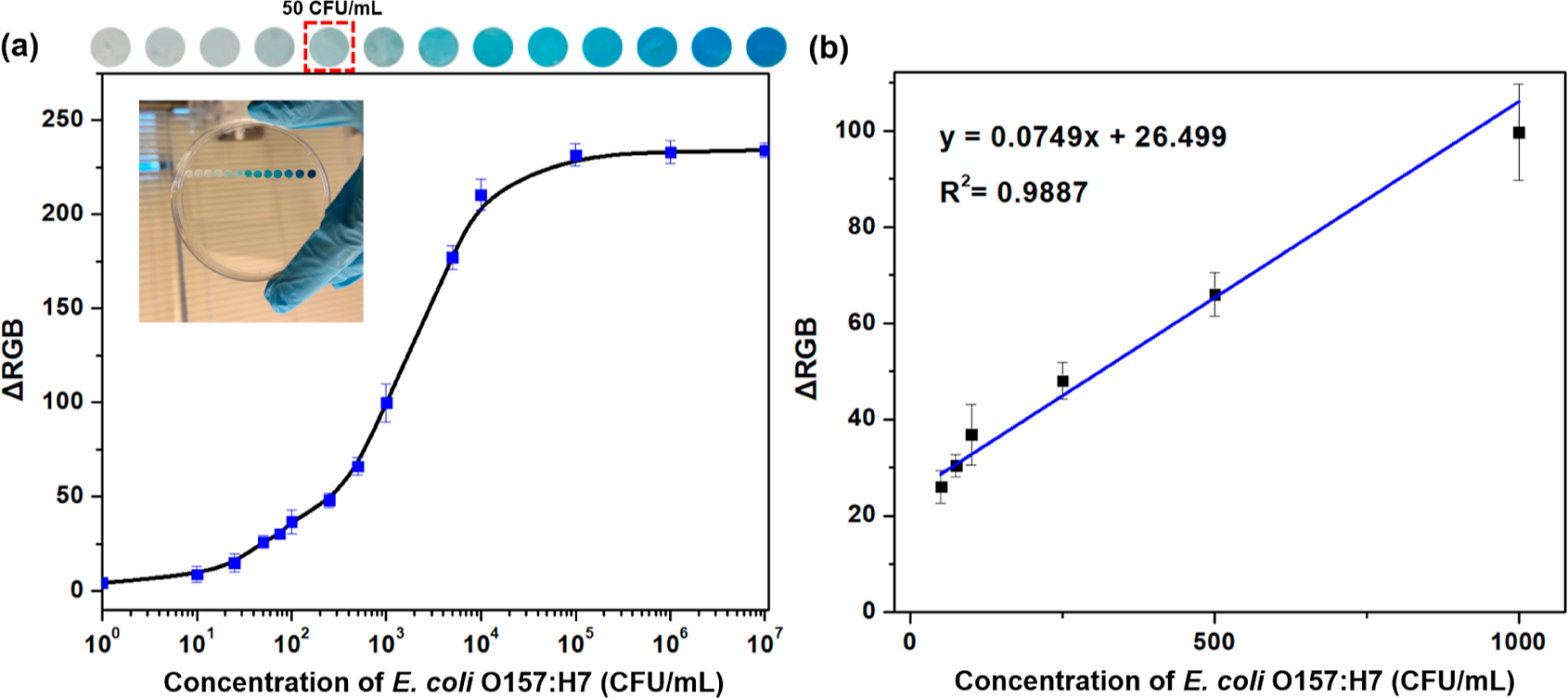
(a) Optical images and the calibration curve of the MF media in the detection of *E. coli* O157:H7. (b) Linear equation for the colorimetric assay was fitted to be *y* = 0.0749*x*+ 26.499 (*R*^2^ = 0.989) between 50 and 10^3^ cfu/mL. Data are shown as mean ± SD, based on *n* = 3 independent experiments.

To assess the sensitivity of f-ELISA for detecting target agents, a detection assay was carried out using 200 μL samples with concentrations of *E. coli* O157:H7 ranging from 0 to 10^7^ cfu/mL. The visually discernible blue color signals in varied intensities correspond to different *E. coli* O157:H7 concentrations, as shown in Figure 4a. Through analysis of the color intensities using Photoshop software and applying eq 1, a linear equation for the colorimetric assay was determined as *y* = 0.0749*x* + 26.499 (*R*^2^ = 0.989) between the bacterial concentrations of 50 to 10^3^ cfu/mL (Figure 4b). Based on Figure 4a, the color signal for *E. coli* O157:H7 at 50 cfu/mL level was naked-eye readable, while a LOD of 10 cfu/mL was achieved using a smartphone-acquired image and further analysis of the image from Photoshop for the f-ELISA sensor.

### Impact of Sample Volumes on Immunoassays

**Figure 5:**
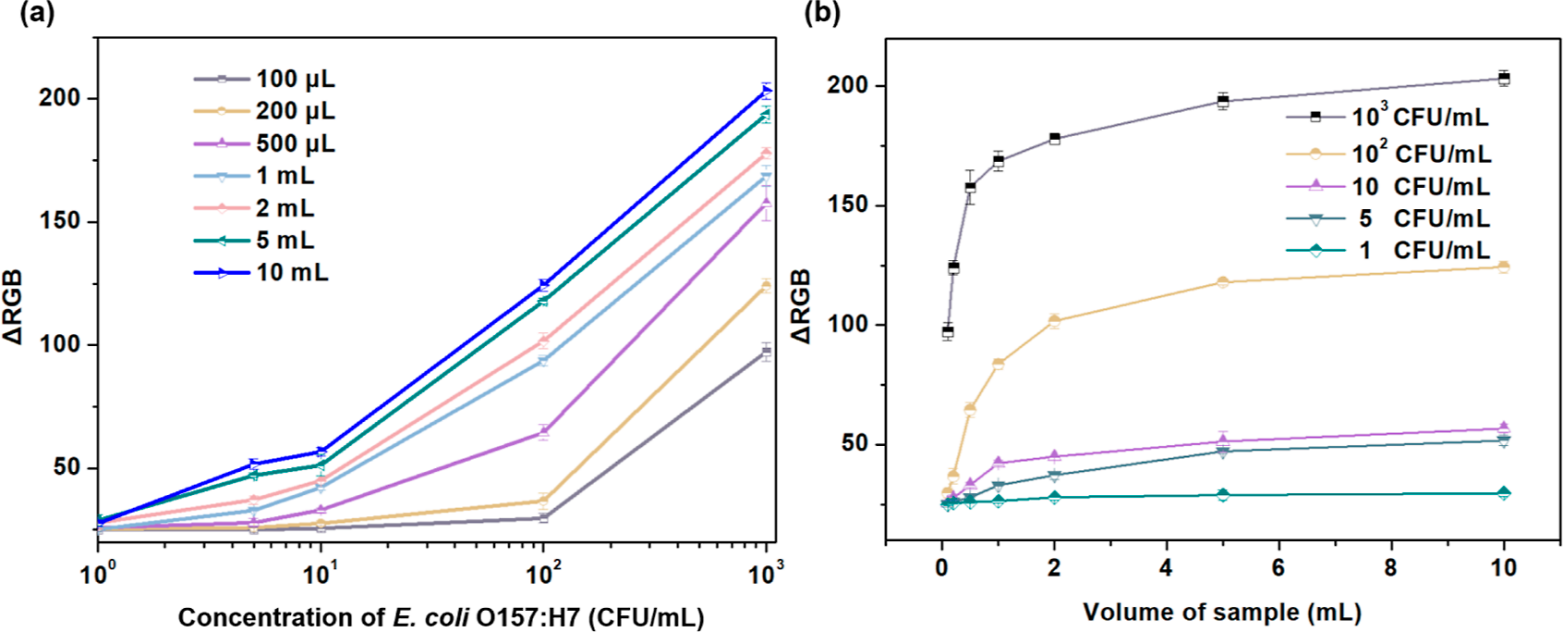
(a) Calibration curves generated using varying sample volumes for the detection of *E. coli* O157:H7. (b) Influence of sample volume on the colorimetric signal intensities in the f-ELISA method. Data are shown as mean ± SD, based on *n* = 3 independent experiments.

Traditional ELISA assays are typically operated within a limited range of sample volumes. Recent innovations, particularly with p-ELISA, have pivoted toward miniaturization, leading to even smaller sample volumes, which are suitable for most biological samples. However, unlike point-of-care clinical analysis, pathogens might exist at very low concentrations in different sources such as various ground and surface water, food samples, and treated industrial wastes. Large volumes of those samples are available and meaningful for the detection of low concentrations of pathogens. Due to the fact that the MF materials in different thicknesses did not lead to a significant increase in fluid resistance (Figure 1) and no nonspecific retention of the targeted microorganism, amplifying the test sample volume allows for a larger number of pathogens binding on the MF structure, thereby increasing the signal intensities. Consequently, even trace amounts of pathogens can be detected when large volumes of test solutions are passed through the foam sensing material. The volume-responsive performances of the f-ELISA were extensively investigated with *E. coli* O157:H7 solution at different volumes: 100 μL, 200 μL, 500 μL, 1 mL, 2 mL, 5 mL, and 10 mL. Apart from the alterations in analyte volumes, all other testing steps were the same as the protocols used in the previous discussions. As shown in Figure 5a, it is clear that different volume sizes produced varying calibration curves. Importantly, when the sample volume was increased from 100 μL to 2 mL, the sensitivity of the f-ELISA in detecting *E. coli* O157:H7 improved significantly, reducing the LOD to 5 cfu/mL (Figure 5b). Due to the unique macroporous structure of the MF, large volume liquid samples can flow without any hindrance, maximizing contact, reactivity, and effective use of the available surface area. Thus, the increase in f-ELISA sensitivity with larger sample volumes can lead to the enhanced probability of antigen–antibody interactions in an increased volume of fluids, creating a stronger signal from accumulated targets. This distinctive structural feature of the f-ELISA sensors makes them ideal for detecting fluid samples in varied volumes and also functioning as a flow-through filtering sensor system for large-volume target solutions with low fluid resistance.

Compared to other sensing materials listed in Table 1, conventional ELISAs using 96-well plates exhibit a LOD of 10^4^ cfu/mL, requiring at least 2 h to complete the tests.^44^ Paper-based ELISAs on Whatman paper reached a LOD of 10^4^ cfu/mL within 3 h.^41^ f-ELISA significantly advanced the field with a LOD of 5 cfu/mL achieved in just 56 min when 2 mL of aqueous sample was tested, making it more sensitive and less time-consuming than most other optical biosensors, as shown in Table 1. These capabilities highlight the suitability of f-ELISA for applications where rapid and highly sensitive on-site detection of bacteria is essential.

**Table 1:** Comparison of Optical Biosensors for the Detection of *E. coli* O157:H7^a^

sensor platform	capture reagent	LOD (cfu/mL)	time	application	reference
Whatman paper	antibody	10^5^	<5 h	urine	(40)
Whatman filter paper	antibody	10^4^	<3 h	lixivium samples	(41)
wax-printed paper	antibody	10^4^	2.5 h	beef samples	(42)
PDA vesicle	antibody	10^4^	2 h	fecal samples and water	(43)
96-well plate	antibody	10^4^	2 h	green tea sample	(44)
GO-Fe_3_O_4_	aptamer	467	30 min	complex biological samples	(45)
iron quantum cluster	amino acids	8.3 × 10^3^	30 min	urine, tap water	(46)
T-bacteriophage	PP0 1ccp phage	1	15 h	apple juice	(47)
functionalized gold NPs	reduce exogenous	10^2^	1 h	complex artificial sepsis blood	(48)
chemically modified MF	antibody	5	56 min	agricultural water and milk	this work
chemically modified MF	antibody	2	2 h	agricultural water and milk	this work

a *LOD = limit of detection, *PDA = polydiacetylene, *GO = graphene oxide.

### Enhanced Sensitivity through Pre-enrichment

**Figure 6:**
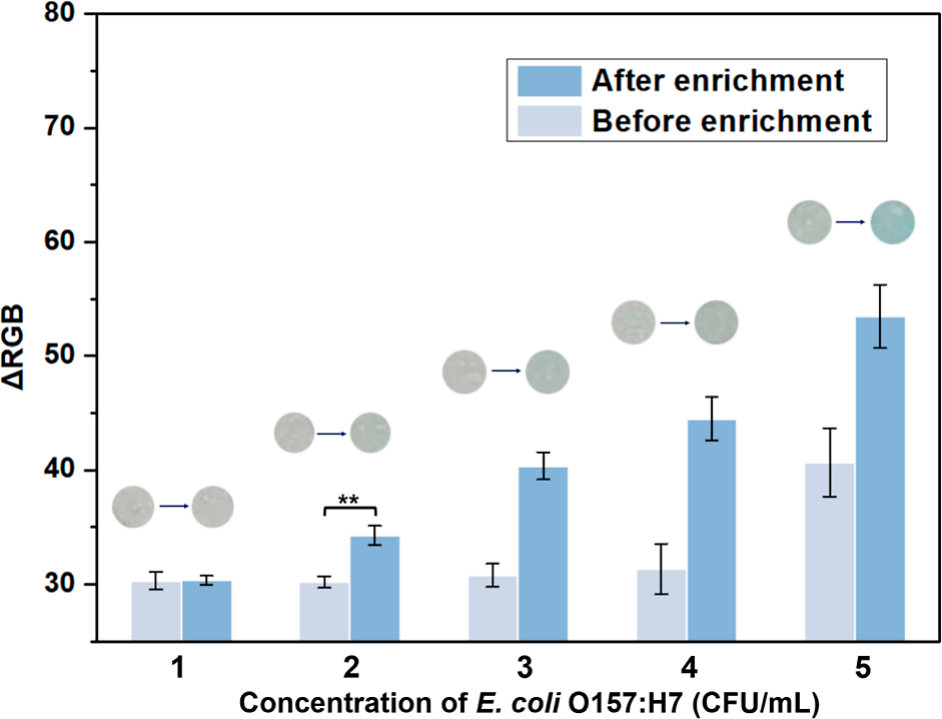
Optical images and the colorimetric signals before and after 1 h enrichment of *E. coli* O157:H7 at various concentrations. Data are shown as mean ± SD, based on *n* = 3 independent experiments. **P < 0.01 (two-tailed Student’s *t*-test).

To further enhance the biosensor’s sensitivity, we incubated the target specimen in the TSB medium for an hour under 37 °C prior to the f-ELISA detection. Consequently, the colorimetric signal of 2 mL of *E. coli* O157:H7 at a concentration of 2 cfu/mL became discernible through analysis of images taken by a smartphone (Figure 6). Even though an extra hour is needed, the overall time taken by the f-ELISA-based biosensor, including incubation, remains under 2 h, a duration that is relatively rapid for detecting bacterial concentrations.

### Selectivity of f-ELISA

**Figure 7:**
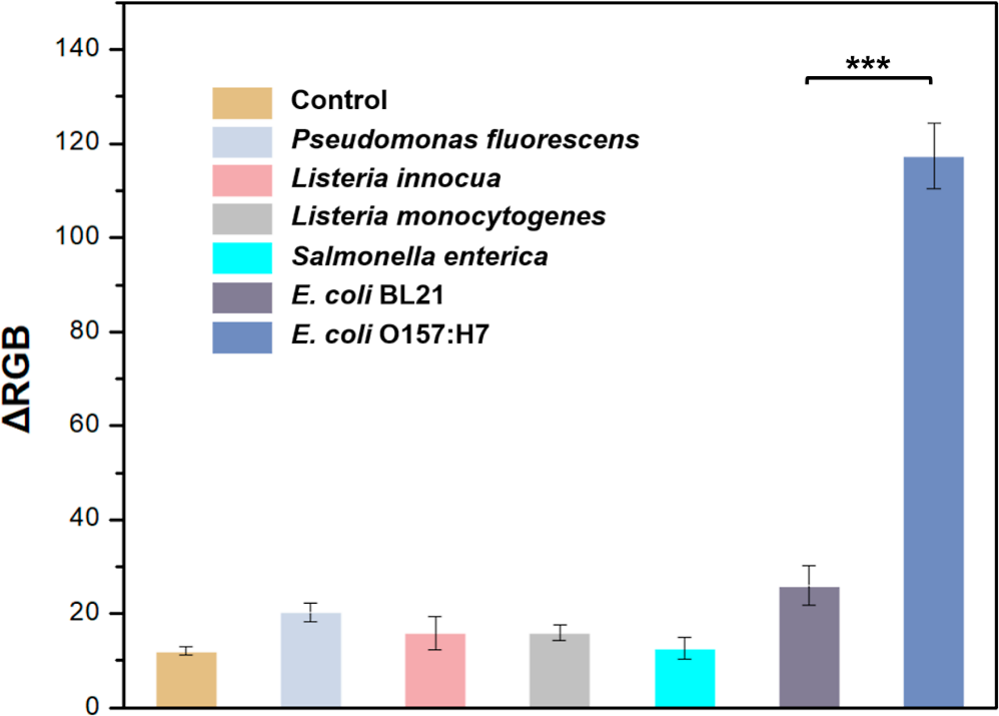
Selectivity of f-ELISA toward *E. coli* O157:H7 detection in comparison with other bacteria strains, including *Pseudomonas fluorescens*, *Listeria innocua*, *Listeria monocytogenes*, *Salmonella enterica*, and *E. coli* BL21. The concentration of each bacterial strain used in this experiment is 10^5^ cfu/mL. Data are shown as mean ± SD, based on *n* = 3 independent experiments. ***P < 0.001 (two-tailed Student’s *t*-test).

The selectivity of the developed f-ELISA biosensing platform was evaluated using various bacterial strains: *Pseudomonas fluorescens*, *Listeria innocua*, *Listeria monocytogenes*, *Salmonella enterica*, and *E. coli* BL21. In our tests, only *E. coli* O157:H7 produced a discernible colorimetric signal, as illustrated in Figure 7. Intriguingly, even a different strain of *E. coli* (BL21) failed to yield a significant signal, underscoring the good selectivity of the immobilized antibody on this biosensor. This specific reaction with only *E. coli* O157:H7 and the nonreactivity with other bacterial strains provides confidence in the applications of the f-ELISA-based biosensor. It offers the potential for accurate pathogen detection in real-world scenarios without being affected by the presence of other bacterial strains.

### Real Sample Analysis

**Figure 8:**
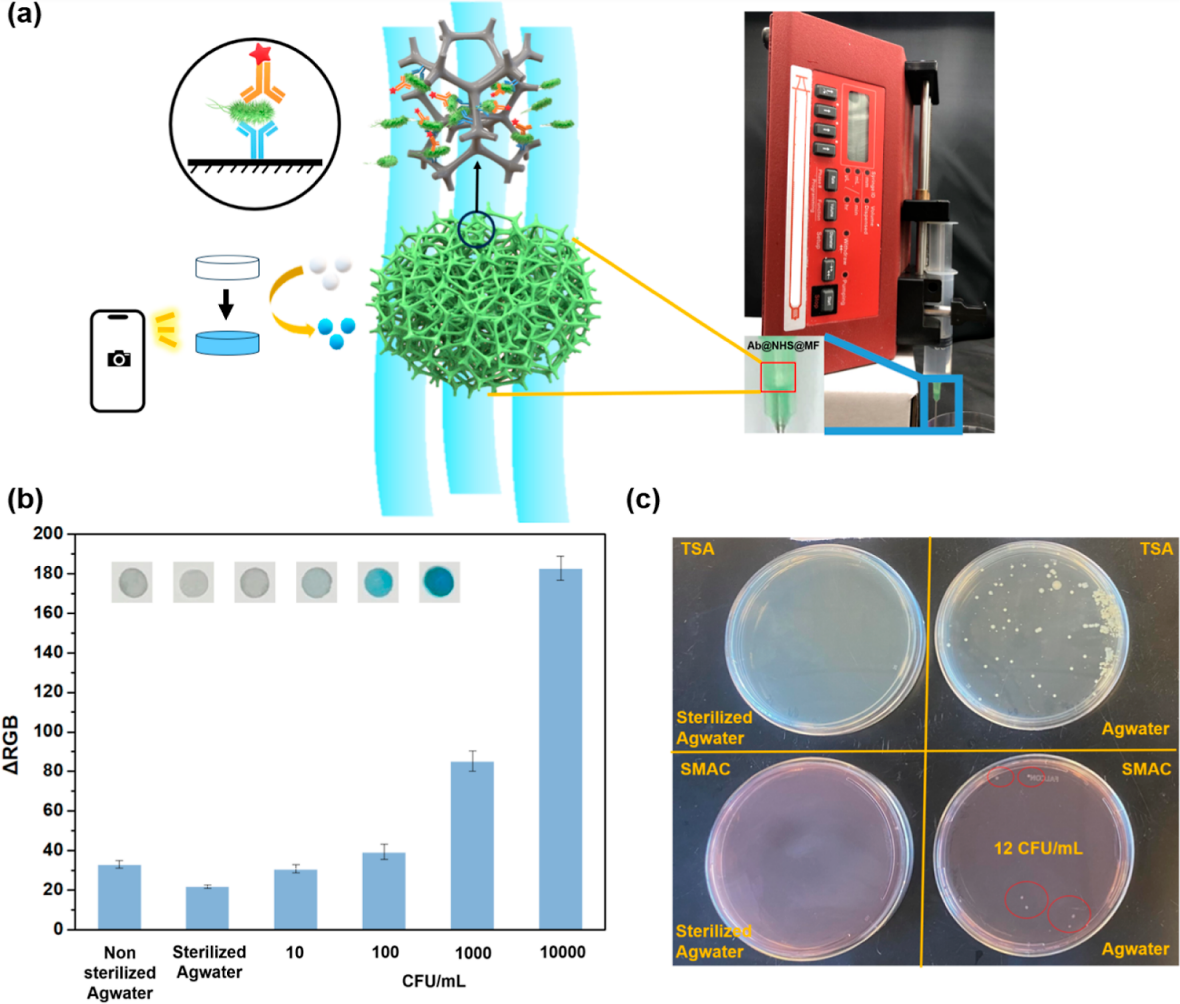
(a) The scheme illustrated the sensing of *E. coli* O157:H7 in agricultural water, and the photograph showcased a rapid-flow device operated by a syringe pump. (b) Optical images and ΔRGB values of membranes treated by different concentrations of *E. coli* O157:H7 in spiked samples, sterilized Agwater, and nonsterilized Agwater. (c) Whole plate images of bacterial cultures upon exposure to sterilized and nonsterilized agricultural water. Data are shown as mean ± SD, based on *n* = 3 independent experiments.

To investigate the efficiency of the f-ELISA in real-world scenarios of *E. coli* O157:H7 detection, we designed artificially contaminated milk samples bought from a local grocery market and agricultural water (Agwater) collected from the irrigation facility at UC Davis, California. The MF membrane was mounted into a syringe needle pocket; 5 mL of a sample solution was loaded into a 20 mL syringe and flowed through the MF in the needle with a controlled flow rate of 10 mL/h (Figure 8a). For each test, *E. coli* O157:H7 was detectable at a concentration of 10 cfu/mL with a sample volume of 5 mL in a flowing-through filtering sensor system demonstrated in Figures 8b and S7. Interestingly, for the nonsterilized agricultural water, the f-ELISA’s colorimetric intensity was just above that of a 10 cfu/mL spiked sample. Subsequent culture plate assays confirmed the presence of *E. coli* O157:H7 at 12 cfu/mL in the agricultural water sample using SMAC as a selective and differential medium for the detection of *E. coli* O157:H7, which aligns with the biosensor results (Figure 8c). The bacterial colonies grown in the TSA medium indicated that there were some other strains of bacteria that existed in Agwater (Figure 8c). This instance effectively confirms the biosensing platform’s specificity and precision. Overall, the results suggest that the developed f-ELISA biosensor is reliable and accurate in detecting *E. coli* O157:H7 in complex matrices.

### Storage Stability Evaluation for f-ELISA

Oxidation, isomerization, and hydrolysis prevent the antibody from existing long-term in the liquid state, diminishing the efficiency of the antibody–antigen interaction.^49^ In contrast, when antibodies are immobilized on a solid phase, they can retain their activity for an extended period.^50^ To ensure the long-term efficacy and repeatability of the f-ELISA system, we examined the stability and activity of the stored f-ELISA biosensing platform. The Ab@NHS@MF membranes were prepared using 10% sucrose as a stabilizer followed by freeze-drying.^51^ They were stored at a consistent temperature of 4 °C and assessed over a period of 90 days. At predetermined time intervals, sample membranes were retrieved and utilized in the f-ELISA assay to detect *E. coli* O157:H7 following the same protocols. The results indicate that the antibodies stored at 4 °C maintained their activity for up to 80 days, showing minimal variation from the results obtained with fresh Ab@NHS@MF membranes (Figure S8). Therefore, with the employment of sucrose as a stabilizer for antibodies, prolonged storage without compromising the efficiency of the f-ELISA system can be achieved.

## Conclusions

The development and evaluation of a novel f-ELISA biosensor for the detection of *E. coli* O157:H7 are presented here. This sensor, constructed with NHS@MF, possessing a macroporous reticulated 3D framework structure, demonstrated high sensitivity, specificity, and selectivity, outperforming other conventional methods presented in the literature. The method required less than 56 min to complete the detection and demonstrated a sensitivity of 10 cfu/mL, with color signals discernible by the naked eye, and an enhanced sensitivity of 5 cfu/mL with the help of a smartphone. Following a brief bacteria enrichment period of 1 h, the sensitivity was further amplified to 2 cfu/mL. Interestingly, the sensitivity increases as the volume of the sample increases, making this method highly suitable for testing large-volume samples, such as milk, agricultural water, etc. In essence, using *E. coli* O157:H7 as a proof of concept, this work not only paves the way for improved bacterial detection in environmental and food samples but also introduces f-ELISA as a new model that could be adapted for other pathogens and contaminants.

## Supporting Information Available

Measurement of diffusion of bacteria in MF; vertical flow test through materials; reagent modification and protein immobilization; optimization experiments for the detection of *E. coli* O157:H7; specificity of the assay; onsite detection of *E. coli* O157:H7 in spiked milk samples; and antibody storage evaluation for f-ELISA (PDF)
