# Supplementary material for: Rapid and Ultrasensitive Colorimetric Biosensors for Onsite Detection of Escherichia coli O157:H7 in Fluids
Source: ACS Sens. 2024 Feb 6;9(2):912–22. doi: 10.1021/acssensors.3c02339 (PMC10897931; doi:10.1021/acssensors.3c02339)
Supplement: Supplementary file 1 — se3c02339_si_001.pdf [file se3c02339_si_001.pdf]

Supporting Information

# Rapid and ultra-sensitive colorimetric biosensors for onsite detection of *Escherichia coli* O157:H7 in fluids

Bofeng Pan[a], Ahmed Y. El-Moghazy[b], Makela Norwood[a], Nitin Nitin[a,b], and Gang Sun[a]*

*Gang Sun: gysun@ucdavis.edu

[a]Biological and Agricultural Engineering, University of California, Davis, CA, 95616, USA

[b]Department of Food Science and Technology, University of California, Davis, CA, 95616, USA

Table of Content

I. Measurement of diffusion of bacteria in MF

II. Vertical flow test through materials

III. Reagent modification and protein immobilization

IV. Optimization experiments for the detection of *E. coli* O157:H7

V. Specificity of the assay

VI. Onsite detection of *E. coli* O157:H7 in spiked mike samples

VII. Antibody Storage Evaluation for f-ELISA

I. Measurement of diffusion of bacteria in MF

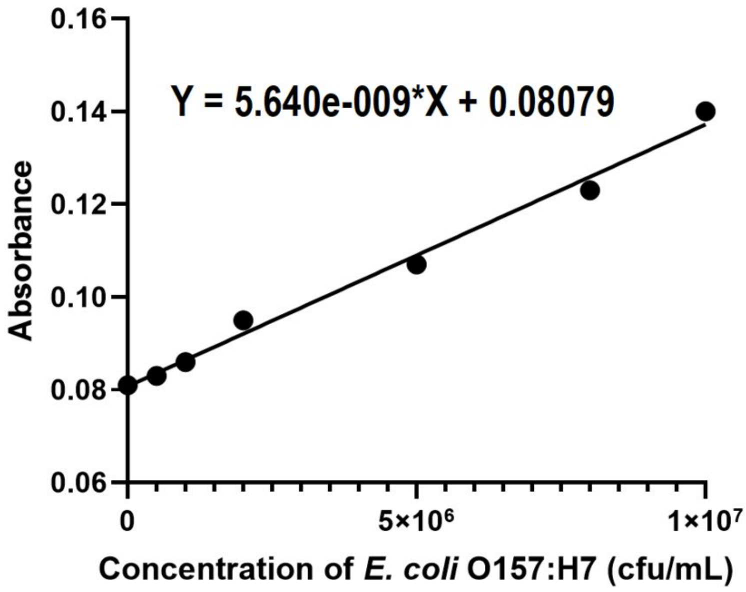


Fig. S1. Calibration curve for *E. coli* O157:H7 at the wavelength of 600 nm.

II. Vertical flow test through materials

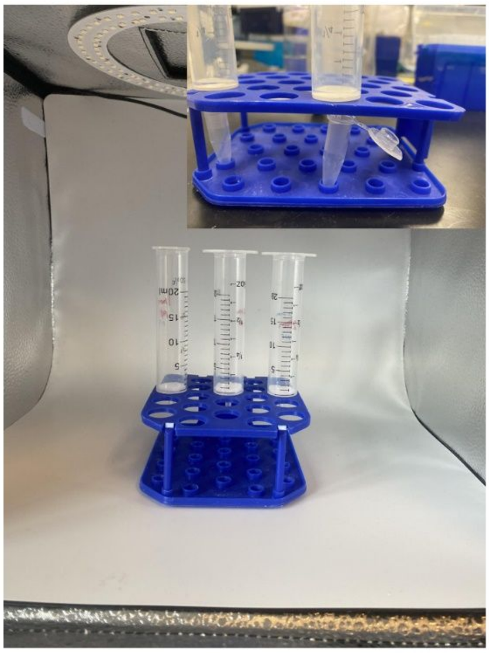

Fig. S2. Photograph demonstrating the liquid filtering test using syringes and vials.

III. Reagent modification and protein immobilization

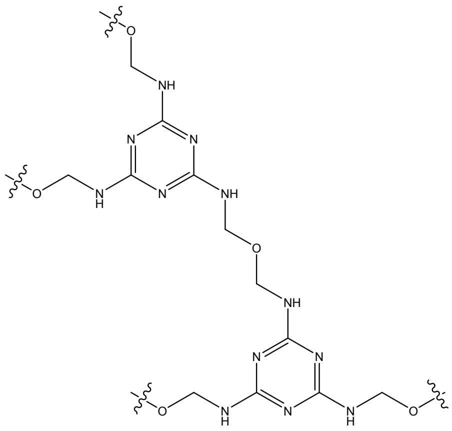

Fig. S3. Chemical structure of melamine foam.

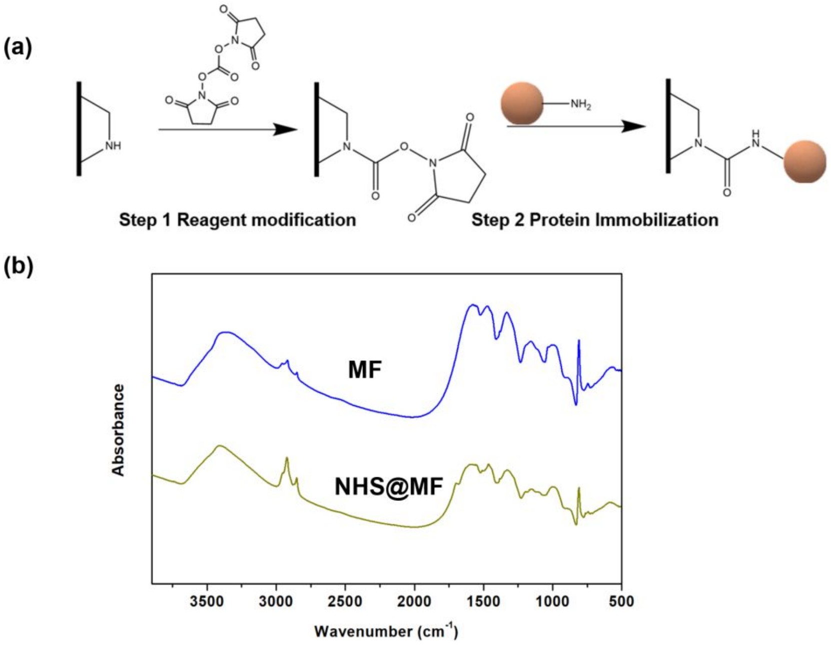


Fig. S4. (a) Reaction of MF with DSC and proteins and (b) FTIR results of MF and NHS@MF.

IV. Optimization experiments for the detection of *E. coli* O157:H7

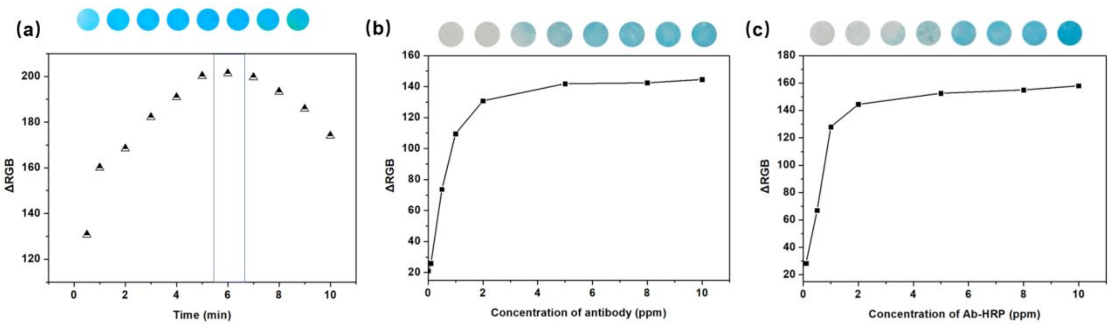


Fig. S5. Optimization of (a) the reaction time between HRP and TMB substrate; (b) the concentration anti-*E. coli* O157:H7 antibodies used for immobilization; (c) the concentration of Anti-*E. coli* O157:H7 antibodies conjugated with HRP used as the secondary antibody in f-ELISA.

V. Specificity of the assay

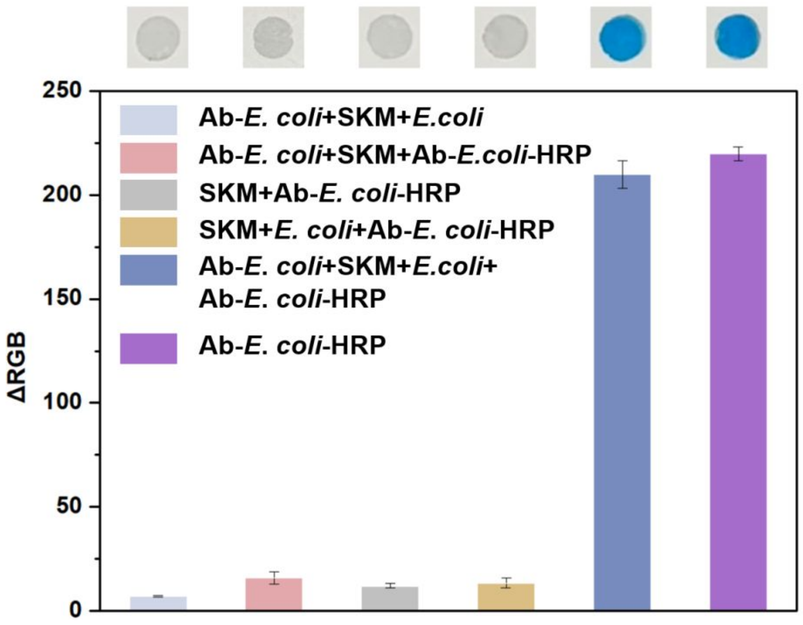


Fig. S6. Specificity of the assay. Images of the NHS@MF membranes with different treatments after the addition of TMB substrate: 100 µL Ab-*E. coli* (5 mg/L), 200 µL skimmed milk (SKM) (3%), 200 µL *E. coli* O157:H7 ($10^7$ CFU/mL), and 100 µL Ab-*E. coli*-HRP (2 mg/L) were used accordingly. The bar diagram for the ΔRGB was

observed from the images. Data are presented as mean ± SD, with n = 3 independent experiments.

VI. Onsite detection of *E. coli* O157:H7 in spiked mike samples

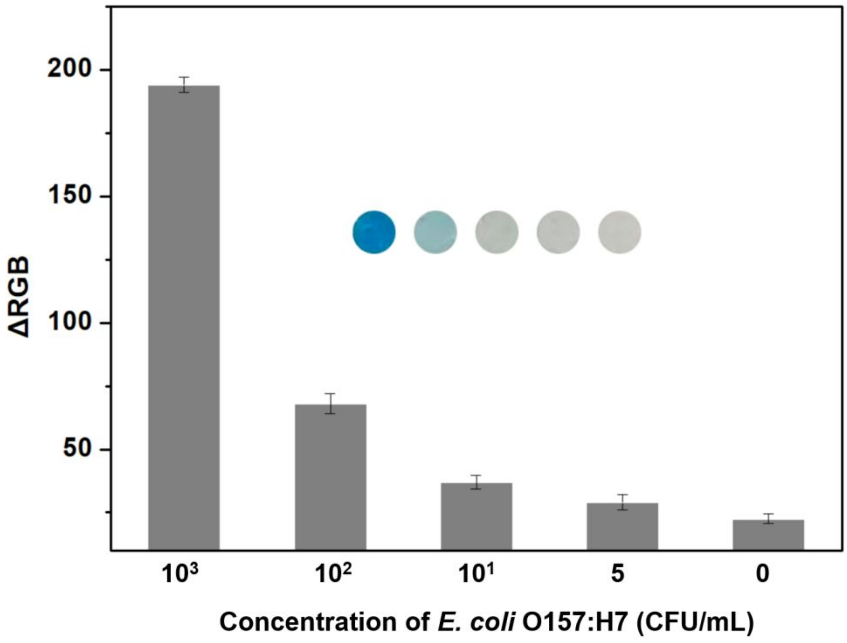


Fig.S7. Optical image and ΔRGB values of membranes treated by different concentrations of *E. coli* O157:H7 in spiked milk samples. Data are presented as mean ± SD, with n = 3 independent experiments.

VII. Antibody Storage Evaluation for f-ELISA

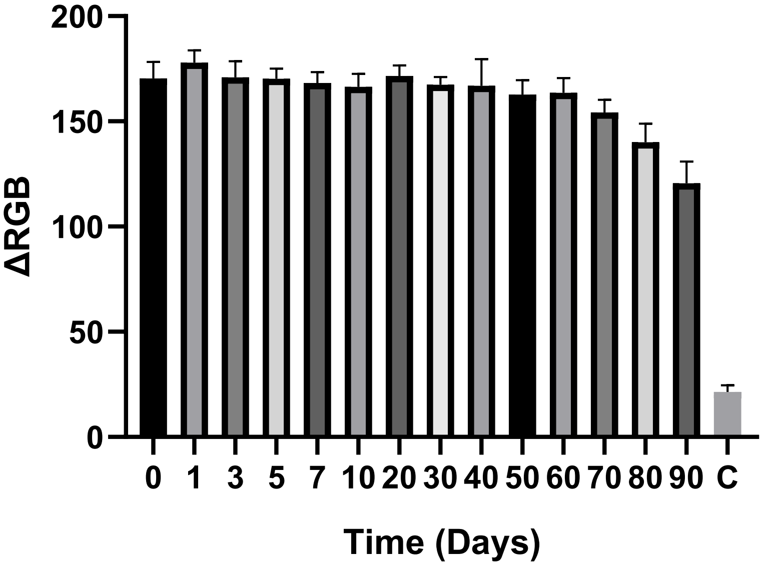


Fig.S8. Long-Term Stability Assessment. The Ab@NHS@MF membranes were prepared by using 10% sucrose as a stabilizer followed by freeze-drying. They were stored at a consistent temperature of 4°C and assessed over a period of 90 days. Data are presented as mean ± SD, with n = 3 independent experiments. *C = Control group
